# Vigilance and Activity Time-Budget Adjustments of Wintering Hooded Cranes, *Grus monacha*, in Human-Dominated Foraging Habitats

**DOI:** 10.1371/journal.pone.0118928

**Published:** 2015-03-13

**Authors:** Chunlin Li, Lizhi Zhou, Li Xu, Niannian Zhao, Guy Beauchamp

**Affiliations:** 1 School of Resources and Environmental Engineering, Anhui University, Hefei, China; 2 Anhui Biodiversity Information Center, Hefei, China; 3 Shengjin Lake National Nature Reserve Management Center, Chizhou, China; 4 Faculty of Veterinary Medicine, University of Montréal, P.O. Québec, Canada; Università della Tuscia, ITALY

## Abstract

Due to loss and degradation of natural wetlands, waterbirds increasingly rely on surrounding human-dominated habitats to obtain food. Quantifying vigilance patterns, investigating the trade-off among various activities, and examining the underlying mechanisms will help us understand how waterbirds adapt to human-caused disturbances. During two successive winters (November-February of 2012–13 and 2013–14), we studied the hooded crane, *Grus monacha*, in the Shengjin Lake National Nature Reserve (NNR), China, to investigate how the species responds to human disturbances through vigilance and activity time-budget adjustments. Our results showed striking differences in the behavior of the cranes when foraging in the highly disturbed rice paddy fields found in the buffer zone compared with the degraded natural wetlands in the core area of the NNR. Time spent vigilant decreased with flock size and cranes spent more time vigilant in the human-dominated buffer zone. In the rice paddy fields, the birds were more vigilant but also fed more at the expense of locomotion and maintenance activities. Adult cranes spent more time vigilant and foraged less than juveniles. We recommend habitat recovery in natural wetlands and community co-management in the surrounding human-dominated landscape for conservation of the hooded crane and, generally, for the vast numbers of migratory waterbirds wintering in the middle and lower reaches of the Yangtze River floodplain.

## Introduction

Human activities have resulted in the losses of over half of the wetlands in the world and extensive degradation of the remnant patches [1,2]. Due to the reduced carrying capacity of natural wetlands, many waterbirds are forced to find extra food in artificial wetlands to meet their energetic requirements [3–5]. How animals adapt to more frequent human-caused disturbances in the artificial wetlands has become the focus of researchers and conservation practitioners [6–8].

Human disturbances in an artificial landscape can be viewed as a form of predation risk, which affects the behavior and survival of foragers [9]. Previous studies have found that behavioral plasticity allows animals to modify their time budget as an initial behavioral response to human disturbances [10–12]. In particular, animals allocate more time to scanning for human disturbances, which can potentially exceed the impact of natural enemies on wildlife behavior [13,14]. As a strategy, maintaining high vigilance can increase the chances of detecting a threat early [15,16]. But high vigilance often reduces the time available for other fitness-enhancing activities such as foraging and resting [17–19]. Quantifying individual vigilance patterns and investigating how animals balance the trade-off between vigilance and other crucial activities can help us understand how animals adapt to human disturbances.

To isolate the effect of human disturbances on individual vigilance patterns, it is necessary to control for the many intrinsic and extrinsic factors that can influence vigilance and might be associated with the level of disturbances. Some potential confounding factors include age [7], sex [20,21], reproductive status [22,23], social rank [24], predation risk [25,26], food density [27] and position in the group [28]. But perhaps the factor that has attracted the most attention is group size. The vast majority of past studies have documented a decrease in vigilance with group size [29,30]. The group-size effect on vigilance reflects the perception of a lower predation risk in larger groups. Individuals in a large group benefit from more eyes and ears to detect approaching threats and experience a dilution of risk caused by the presence of alternative targets in the group for the predator [31].

As a migratory wading bird, the hooded crane *Grus monacha* breeds in south-central and south-eastern Siberia, Russia and Heilongjiang, north-eastern China and winters in Japan, South Korea and south-eastern China. Due to wetland losses and degradation, the species is in sharp decline, and is listed as Vulnerable on the IUCN Red List and Category I Key National Protected Wild Animal Species in China [32]. One tenth of the global population that winters in China is limited to the ephemeral wetlands of the middle and lower Yangtze River floodplain [33]. The natural habitats in the region are degrading through a combination of wetland reclamation, aquaculture-induced collapse of submerged macrophyte dominated vegetation community and dam-caused hydrological discontinuity with the Yangtze River [34,35]. Reduced carrying capacity in the natural wetlands has made the surrounding rice paddy fields an important source of food for the wintering cranes [36,37]. Human disturbances originating from agricultural activities, however, are frequent in the paddies, and might have profound effects on crane’s behaviors and survivorship. Thus far, little is known about how the species responds to changes associated with foraging habitat shift, which is important to know as a first step to inform future conservation plans.

In this study, we investigated how the hooded cranes adapt their vigilance and activity time budget to human disturbances in the artificial wetlands. To this end, we took behavioral samples both in the buffer zone and the core area of the Shengjin Lake National Nature Reserve (NNR). The core area of the NNR covers the open water area of the lake with reduced food for cranes but low levels of human disturbances in the wintering period [34,37]. The buffer zone is dominated by low-lying agricultural fields where rice and oil-seed rape are grown. The hooded cranes favor grains left over after harvest in the rice paddies, but their foraging is frequently interrupted by human activities. We surmised that cranes would adapt their vigilance patterns to different levels of human disturbances. We predicted that cranes foraging in the buffer zone would spend more time vigilant to detect threats more rapidly. We contrasted vigilance in the two areas controlling for flock size and age, which are two main factors influencing vigilance in other crane species [6,28,38]. We also looked at other components of the activity time budget, such as foraging and locomotion, to determine if the cranes modify other activities in a human-dominated environment. Based on the results, management recommendations are proposed to further the conservation of the species in the region.

## Methods

### Ethics Statement

We adhered to the “Guidelines for the use of animals in research” published in *Animal Behaviour*. Our research protocols have been approved by the Chinese Wildlife Management Authority. The access to the study sites was approved by the Shengjin Lake National Nature Reserve Management Center. To avoid possible disturbances from observers in the field, we only videotaped (video camera: Sony HDR-CX510, 55× extended zoom, Sony Corporation, Tokyo, Japan) cranes at hidden points c. 200 m away and supplemented our observations with telescope (20–60× zoom Swarovski: ATM 80) and binoculars (10×42 WB Swarovski). The study was observational in nature and caused no undue harm to the birds. No review from the ethics committee was required in China. All the work was conducted under the Wildlife Protection Law of the People’s Republic of China.

### Study area

**Fig 1:**
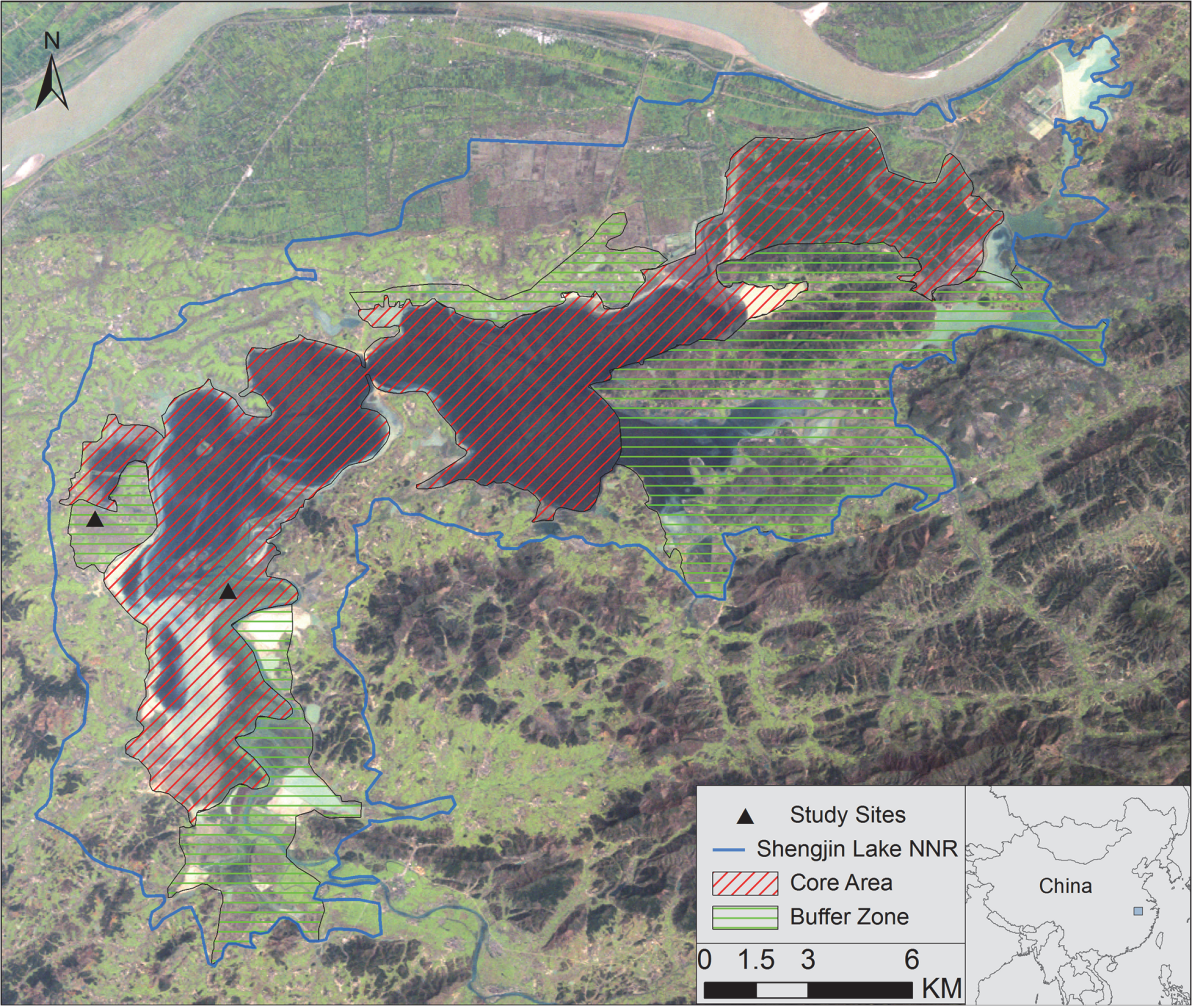
Survey sites. During two successive winters (November-February 2012–13 and 2013–14), we studied vigilance and activity time budget of the hooded crane in both the buffer zone and the core area of the Shengjin Lake National Nature Reserve (NNR), China.

The study was carried out in the upper part of Shengjin Lake (30°15’–30°30’N, 116°55’–117°15’E), which is located on the south bank of middle and lower reaches of Yangtze River (Fig. 1). The Shengjin Lake NNR was established in 1986 and upgraded to a national status in 1997. It joined the Northeast Asia Crane Network Protection Zone in 2002 and the East Asian—Australasian birds Migration Network in 2010. The total reserve covers c. 33,340 ha and the core area encompasses the entire lake area, which reaches a maximum area of c. 14,000 ha in summer. Some 4,000 ha of the riparian mudflat and meadow are exposed during November to March when the water level falls. The average annual rainfall is 1,600 mm, with most falling from March to August. The average annual temperature is 16.1°C, with an average January temperature of 4.0°C.

Shengjin Lake is of global importance for providing essential nesting, staging and migratory habitat for wildfowl along the East Asia-Australasian Flyway [39]. The seasonally inundated wetlands support around 5–10% of the total waterbirds population within the Yangtze River floodplain, including globally threatened wading birds such as Siberian crane *G*. *leucogeranus*, oriental stork *Ciconia boyciana*, swan goose *Anser cygnoides*, hooded crane and white-naped crane *G*. *vipio* [40,41]. The wetlands provide essential wintering habitats for the hooded crane with 300–400 individuals documented in winter [42,43]. Natural predators of the cranes are rare in the NNR given the large body size of the species.

There are c. 100,000 people in total in the area with more than 900 fishermen living in the six towns and 37 administrative villages around the lake. Local residents rely mainly on agriculture and aquaculture [34,39]. Surrounding the upper lake are the scattered settlements and reclaimed agricultural fields where rice and cotton are grown in summer and oil-seed rape in winter. Post-harvested fallen rice grains in the unplowed fields provide important food for the hooded crane [36]. The year-round human activities in the buffer zone include cultivating, harvesting and daily routines. Aquaculture-induced collapse of submerged macrophyte dominated vegetation community resulted in significant food decline in the core area for the hooded crane which preferred tubers of submerged macrophyte, e.g., *Vallisneria* [34]. Concentrated fishing period in the lake ends before wintering waterbirds arrive, reducing human disturbances in the core area of the NNR.

### Behavioral Observations

We carried out field data collection from sunrise (0700) to sunset (1730) during two successive winters from November to February (11 days in 2012–13 winter and 17 days in 2013–14 winter). We made observations on sunny days without strong wind or heavy fog. Focal flocks of the hooded crane were selected as they were encountered along fixed survey routes, which were not repeated on the same day. We defined a flock as a collection of individuals all occurring within 30 m of one another, visually estimated using pre-measured distances between nearby landmarks. Beyond this distance, individuals showed little coordination of activities.

We videotaped (video camera: Sony HDR-CX510, 55× extended zoom, Sony Corporation, Tokyo, Japan) focal flocks and supplemented our observations with telescope (20–60× zoom Swarovski: ATM 80) and binoculars (10×42 WB Swarovski). While taking videos, contextual information about each flock, including flock composition, date, time, habitat and human disturbances were recorded. Disturbances were defined as human activities occurring within 200 m of the cranes and were quantified as the number of occurrences per hour. Videotaping was stopped when 1) the target flock composition changed, 2) any flock member could not be visually followed, 3) the flocks scattered too much to allow the camera to clearly capture the whole flock, and 4) human activities disturbed the flocks. To avoid possible disturbances from the initial approach of the observers, videotaping was made at hidden points (e.g., behind bushes or slopes) c. 200 m away from the cranes, measured with a ranger finder (Nikon 1200S with measurement range of 10–1,100 m). Furthermore, the observers waited several minutes prior to videotaping. Each flock was typically videotaped for about 15 min. We discarded video records shorter than 1 minute to increase data reliability and representativeness, as was suggested in other studies [22,44]. After exclusion, we obtained 245 video records (with an average of 8.8 per day), which we used for data collection.

We used focal sampling to record individual behavioral patterns from each videotape. The same observer watched all videotapes after extensive training. In each flock, one juvenile (if present) and one to two non-contiguous adult cranes were randomly selected as focal individuals. Cranes were not marked and could not be individually recognized. Individuals on the screen were numbered from left to right, and focal cranes were then selected using a random number generator. Considering the local population size (300–400) [42,43] and our use of a random selection procedure, we believe that pseudoreplication was not a major issue in this study. In total, we obtained 601 focal samples amounting to 4,808 min over the two winters. Among these, 368 observations (61.2%) were carried out in the core area and the rest in the buffer zone. Sampling durations lasted from 1 to 21.6 min, with an average of 8 min (Table 1).

**Table 1:** Summary of focal observation samples of the hooded crane in the Shengjin Lake NNR, China.

Area	Age	Number of observations	Total observation time (min)	Average group size	Range of group size
2012–2013 winter					
In core area	Adults	27	205.4	5.2 ± 2.9	1–40
	Juveniles	7	35.3		
In buffer zone	Adults	35	251.6	16.9 ± 3.0	4–33
	Juveniles	13	95.9		
2013–2014 winter					
In core area	Adults	248	2008.0	4.7 ± 0.5	1–34
	Juveniles	86	675.5		
In buffer zone	Adults	137	1133.6	11.5 ± 2.6	2–109
	Juveniles	48	403.0		

We defined crane behavioral patterns following Zhou et al. [37], i.e., foraging, vigilance, locomotion, maintenance and other behaviors. Foraging consists of probing, pecking, excavating and swallowing food. Vigilance occurs when a crane stretches the head upwards while scanning around or gazing at a fixed location. Locomotion includes walking, running, leaping and flying without vigilance. Maintenance includes resting, preening, head shaking, leg stretching and fluttering. Behaviors that were not listed in the above categories were labeled as other behaviors. During focal sampling, cranes were divided into adults and first-winter juveniles. Juveniles were easily distinguished by their brownish or grayish feathers on the head and neck [33,45]. Sex of the cranes was ignored because it cannot be distinguished in the field.

### Data Analysis

We calculated three vigilance components from the timed behavioral sequences. As a proxy of vigilance used in many other studies [22], time spent vigilant represented the percentage of a focal observation allocated to vigilance. To investigate how cranes actually achieved a given level of vigilance, we also calculated scan rate, the number of vigilance bouts initiated per minute, and mean scan duration, which was calculated by dividing total time spent vigilant by the number of vigilance bouts. Prior to statistical analyses, time spent vigilant was arcsine-square-root transformed, and mean scan duration and flock size were log_10_ transformed to fit the assumption of normality of residuals. A two-sample Student’s *t*-test was used to compare the log_10_ transformed flock size between the core area and the buffer zone. Chi-square goodness-of-fit test was used to examine the difference of the sampled adult-juvenile ratio between the two winters.

For the analysis of time spent vigilant and mean scan duration, we fitted a linear mixed model including year (2012–13 winter vs. 2013–14 winter), age (adult vs. juveniles), area (core area vs. buffer zone) and flock size (continuous variable) as fixed factors, and flock id as a random factor. We first included in the model all the main effects of the explanatory variables and all the two-way interactions. Non-significant effects (*p*>0.05) were removed in the final model by backward elimination.

The distribution of the number of scans per min was heavily right-skewed. In this case, we fitted a mixed negative binomial regression model including the same fixed and random effects as mentioned above. The dependent variable was the number of scanning bouts in a focal observation. The natural logarithm of observation duration (min) was included in the model as an offset because focal observation duration varied among samples. The ratio of the deviance to degrees of freedom was close to 1, indicating a good fit for the negative binomial model.

For each crane, we also calculated the percentage of time allocated to foraging, locomotion and maintenance. Percentage time spent foraging was arcsine-square-root transformed and a linear mixed model was fitted to test the effects of the same factors above. Additionally, we fitted a generalized linear model with a gamma error structure and a logarithmic link function to the heavily right-skewed percentage time spent in locomotion and maintenance. The deviances were also approximately equal to the degrees of freedom, indicating a good fit of the models to the data. Since the time allocated to the different behavioral patterns all add up to 100%, the above time-budget analyses are not technically independent and some of the significant variables might appear in more than one model.

All the statistical procedures were carried out with SAS v 8.1 (SAS Institute Inc., Cary, NC, USA) with statistical significance set at *p*<0.05. Means and SE are reported below.

## Results

### Sampling information

In total, 245 flocks of the hooded crane were videotaped in the field from which we obtained 601 focal timed behavioral sequences. The sampled adult-juvenile ratio did not differ between the 2012–13 winter (3.1:1) and 2013–14 winter (2.9:1) (*χ* ^2^ = 0.076, *p* = 0.783). The main types of human disturbances in the buffer zone included motorcycling, passers-by, car driving and ploughing. The overall rate of disturbances was 8.3 times per hour (0~31.2, *N* = 96). We recorded only 6 occurrences of cattle herding (without pastoralists) and 2 occurrences of passers-by within 200 m of the focal flocks in the core area. Flock sizes were larger in the buffer zone than in the core area (*t* = 6.18, *p*<0.001; Table 1).

### Vigilance components

**Fig 2:**
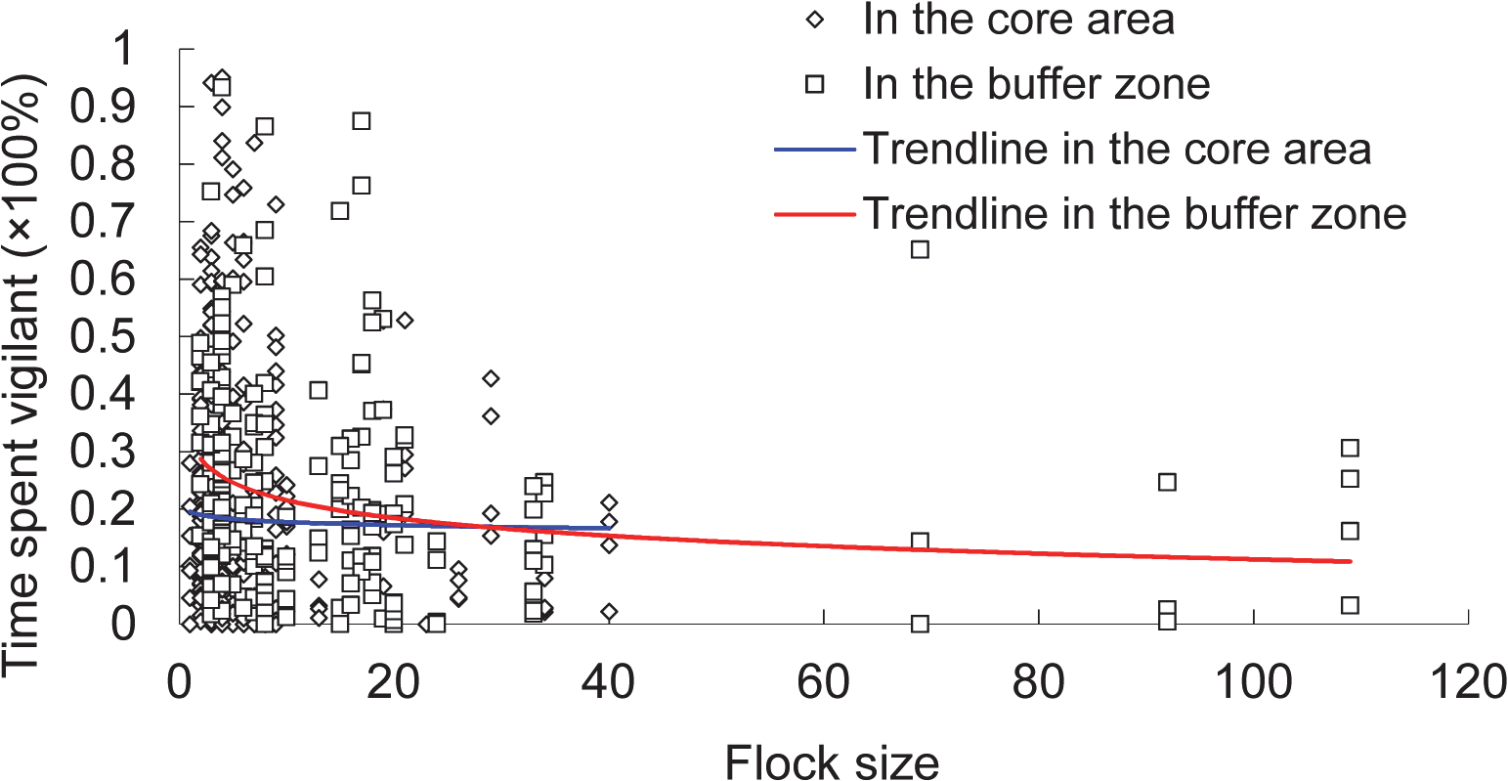
Percentage time spent vigilant as a function of flock size of the hooded crane in the core area A) and the buffer zone B) of the Shengjin Lake NNR, China.

The percentage time spent vigilant ranged from 0 to 95.1% (Table 2). The final linear mixed model revealed a statistically significant effect of year (*F*_*1*,*207*_ = 7.84, *p* = 0.006), age (*F*_*1*,*412*_ = 71.31, *p*<0.001), area (*F*_*1*,*219*_ = 10.87, *p* = 0.001), flock size (*F*_*1*,*205*_ = 6.76, *p* = 0.010) and the two-way interaction between area and flock size (*F*_*1*,*204*_ = 4.80, *p* = 0.030). The hooded crane spent more time vigilant in 2012–13 winter (25.8% ± 2.6%) than in 2013–14 winter (18.9% ± 0.8%). Adult cranes spent more time vigilant than juveniles in both the buffer zone (24.4% ± 1.4% vs 15.5% ± 2.2%) and the core area (20.3% ± 1.2% vs 12.8% ± 1.7%) in the two winters. Both adult and juvenile cranes spent more time vigilant in the buffer zone than those in the core area. Individual vigilance decreased with flock size (*β* = −0.158 ± 0.036, *t* = −4.36, *p*<0.001) in the buffer zone but not in the core area (*β* = −0.050 ± 0.044, *t* = −1.13, *p* = 0.260) (Fig. 2).

**Table 2:** Activity time budget of the hooded crane wintering in the Shengjin Lake NNR, China.

Area	Age	Foraging	Vigilance	Locomotion	Maintenance
2012–2013 winter					
In core area	Adults	45.8% ± 6.4%	34.7% ± 4.6%	8.7% ± 1.7%	10.7% ± 3.9%
	Juveniles	38.3% ± 14.9%	29.5% ± 8.3%	9.3% ± 3.6	21.9% ± 10.2%
In buffer zone	Adults	70.0% ± 5.8%	20.3% ± 3.8%	0.8% ± 0.3%	8.8% ± 3.8%
	Juveniles	73.0% ± 7.4%	20.4% ± 5.8%	1.3% ± 0.8%	5.0% ± 2.4%
2013–2014 winter					
In core area	Adults	31.7% ± 1.7%	18.8% ± 1.2%	32.2% ± 1.7%	16.8% ± 1.7%
	Juveniles	47.2% ±3.3%	11.5% ± 1.7%	27.8% ± 2.5%	12.7% ± 2.5%
In buffer zone	Adults	70.7% ± 1.7%	25.4% ±1.4%	2.1% ± 0.4%	1.7% ± 0.6%
	Juveniles	84.4% ± 2.4%	14.1% ± 2.3%	0.8% ± 0.2%	0.7% ± 0.3%

**Fig 3:**
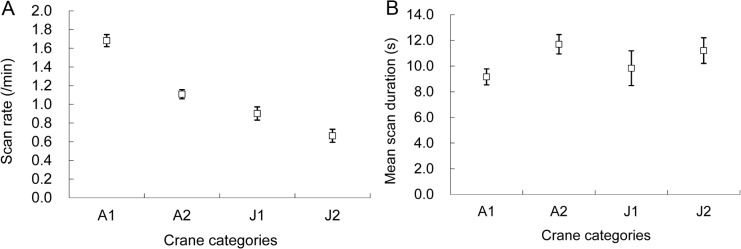
Scan rate A) and mean scan duration B) of the hooded crane wintering in the Shengjin Lake NNR, China: A1, adult cranes in the buffer zone; A2, adults in the core area; J1, juveniles in the buffer zone; J2, juveniles in the core area.

Mean scan duration ranged from 0.4 s to 80.0 s. The final linear mixed model indicated no significant effect of year (*F*_*1*,*187*_ = 0.45, *p* = 0.506), age (*F*_*1*,*388*_ = 0.49, *p* = 0.486), area (*F*_*1*,*183*_ = 1.31, *p* = 0.253) or flock size (*F*_*1*,*184*_ = 1.51, *p* = 0.220) on mean scan duration. Scan rates ranged from 0 to 5.44 min^−1^. The final negative binomial regression model showed a statistically significant effect of year (*χ* ^*2*^ = 6.56, *p* = 0.010), age (*χ* ^*2*^ = 94.26, *p*<0.001), area (*χ* ^*2*^ = 80.90, *p*<0.001) and flock size (*χ* ^*2*^ = 16.10, *p*<0.001). The hooded crane scanned more frequently in 2012–13 winter (1.35 ± 0.09 min^−1^) than in 2013–14 winter (1.16 ± 0.04 min^−1^). Adult cranes scanned more frequently than juveniles in both the buffer zone (1.68 ± 0.06 min^−1^ vs 0.90 ± 0.07 min^−1^) and the core area (1.11 ± 0.05 min^−1^ vs 0.66 ± 0.07 min^−1^). Scan rate decreased with the flock size (*β* = −0.007 ± 0.002) and the cranes scanned more frequently in the buffer zone than in the core area (Fig. 3).

### Other activities

Wintering hooded cranes allocated the majority of their time (99.6% ± 0.2%) to foraging, vigilance, locomotion and maintenance. Time spent foraging represented the largest component of the time budget for both adult and juvenile cranes in the two areas (Table 2). For time spent foraging, the final linear mixed model showed a significant effect of age (*F*_*1*,*405*_ = 74.94, *p*<0.001), area (*F*_*1*,*216*_ = 5.78, *p* = 0.017), and a two-way interaction between area and flock size (*F*_*2*,*202*_ = 3.16, *p* = 0.044) but no effect of year (*F*_*1*,*203*_ = 0.03, *p* = 0.868). Juvenile cranes spent more time foraging than adult cranes in both the buffer zone (81.9% ± 2.5% vs 70.5% ± 1.8%) and the core area (46.5% ± 3.2% vs 33.1% ± 1.7%). Both adult and juvenile cranes increased time foraging in the buffer zone compared with the core area. Time spent foraging increased with flock size in the buffer zone (*β* = 0.182 ± 0.046, *t* = 3.96, *p*<0.001), but there was no significant effect of flock size in the core area (*β* = −0.030 ± 0.063, *t* = −0.48, *p* = 0.633).

For locomotion, the final generalized linear model revealed significant effect of year (*χ* ^2^ = 30.55, *p*<0.001), area (*χ* ^2^ = 337.00, *p*<0.001), flock size (*χ* ^2^ = 15.20, *p*<0.001) but not age (*χ* ^2^ = 3.03, *p* = 0.082) on percentage time spent on locomotion. The hooded crane spent more time on locomotion in the 2013–14 winter (20.6% ± 1.1%) than in the 2012–13 winter (4.2% ± 0.8%). Both adult and juvenile cranes reduced time spent on locomotion in the buffer zone compared with the core area. There was a negative significant effect of flock size on time spent on locomotion (*β* = −0.016 ± 0.004). Percentage time spent on maintenance was significantly influenced by year (*χ* ^2^ = 10.28, *p* = 0.001) and area (*χ* ^2^ = 65.80, *p*<0.001) but not age or flock size (*p*>0.05). The cranes spent more time on maintenance in the 2013–14 winter (10.6% ± 1.0%) than in the 2012–13 winter (9.9% ± 2.3%). Both adult and juvenile cranes spent more time on maintenance in the core area (Table 2).

## Discussion

Our observations revealed that the level of human disturbances was much higher in the buffer zone than in the core area of the Shengjin Lake NNR. Therefore, cranes faced a choice between foraging in a rich feeding area but at the risk of being disturbed more frequently and foraging in a food-poor area with less frequent disturbances. Overall, we found a significant effect of year, area, age and a strong negative effect of flock size on vigilance behavior in cranes, as found in many other species [29,30]. Cranes spent more time vigilant but less on locomotion in the 2012–13 winter than in the 2013–14 winter. However, the year effect is difficult to interpret given that there were no obvious differences between the two successive winters in weather, rates of disturbances or foraging contingencies. We focus below on the other independent variables.

Both adult cranes and juveniles spent more time vigilant in the artificial wetlands in the buffer zone than in the core area. In addition to the level of human disturbances, many other factors might also contribute to the observed difference in vigilance patterns between the two areas. Flock size, for one, was typically larger in the buffer zone, but we documented a difference in vigilance after controlling for this factor. Other environmental variables such as visibility [46,47], predation risk [25,48], and distance to cover [49,50] are less relevant for a large species like the hooded crane with few predators and foraging in a largely open habitat. However, food density certainly differed between the two areas [34,36,37], which might modulate the group-size effect on vigilance. When foragers face time constraints, vigilance is predicted to decrease with food density [27]. Assuming that cranes faced such constraints and that the buffer zone provides more food per unit time spent foraging, which is reasonable because cranes fed there on abundant food leftovers [37,51], vigilance should be lower in the buffer zone than in the core area. Controlling for flock size, we documented the opposite pattern from which we conclude that the level of human disturbances was the primary factor affecting the difference in vigilance between the two areas.

In a human-dominated landscape, anthropogenic disturbance has become one of the key components of habitats and has been consistently found to increase prey’s time allocation in vigilance [35,52]. This is also the case for the hooded crane wintering in the Yangtze River floodplain. Over the last 40 years, and especially in the last 20 years, natural wetlands in the region have been decreasing through land conversion to agriculture (mainly to rice paddy) [35,39]. Due to collapse of the preferred natural *Vallisneria* beds in the ephemeral mudflat inside the core area of the Shengjin Lake NNR, the hooded crane has notably moved to the surrounding rice paddy fields, which have become important alternative foraging habitats for the species [36]. Intensive human disturbances in the artificial wetland, however, frequently interrupt cranes’ foraging and raise vigilance levels, as found at stopover sites in north-eastern China [35]. Compared with the disturbed buffer zone, human disturbances in the core area in winter are not so common because the concentrated fishing there ends before wintering waterbirds arrive. Furthermore, the wide (>70 m) and deep river along the eastern lakeside reduces human activities in the exposed mudflat and wetland meadow where the cranes wander. The relatively safer environment partly allows a reduction of vigilance.

In both the buffer zone and the core area, adult cranes spent more time vigilant and less time foraging than their first-year young. A similar age effect has been found in other large migratory birds such as sandhill crane *G*. *Canadensis* [53], common crane *G*. *grus* [38], black-necked crane *G*. *negricollis* [54], red-crowned crane *G*. *japonensis* [7] and greater flamingo *Phoenicopterus roseus* [55]. Age-dependent differences in time spent vigilant and foraging are generally attributed to the lack of experience of younger animals with predators and their higher nutritional needs [56]. Juveniles can benefit from the higher vigilance level shown by their parents to detect threats earlier. During the first winter, a greater allocation of time to foraging probably helps juveniles to meet the energetic requirements for physical development and long-distance migration. Interestingly, juveniles showed the same adjustments to disturbances as their parents despite presumably having less experience. It might be the case that juveniles copy the vigilance patterns adopted by their parents. Vigilance copying has been documented in many species [57–60].

Consistent with Zhou et al. [37], the hooded crane spent a large amount of time in vigilance and foraging, which together accounted for over 70% of the total time budget. While moving to the rice paddies, adult cranes increased time spent vigilant predominantly through changes in scan rate rather than scan duration. Such strategy has also been found in other species [6,61]. Animals probably increase scan rate to detect frequent disturbances [62]. In the total time budget, time spent vigilant and foraging both increased in the buffer zone at the expense of locomotion and maintenance. This strategy allows the hooded crane to maintain safety while reducing total time exposed to disturbances. The more spatially concentrated food in the buffer zone allows cranes to reduce time spent on locomotion.

Overall, our results indicate that intensive human disturbance has a profound effect on the behavior of the hooded crane. To survive in the human-dominated wintering ground, cranes adjusted their vigilance pattern and activity time budget. In the light of our results, we make the following conservation recommendations for the hooded crane and, more generally, for the migratory waterbirds wintering in the middle and lower reaches of the Yangtze River floodplain. Firstly, we recommend recovery of the natural submerged macrophyte vegetation community in the ephemeral riparian wetlands and the continuity between the Shengjin Lake and the Yangtze River. By these initiatives, the carrying capacity of the lake should be improved and support more wintering waterbirds. Secondly, community co-management should be a good strategy to protect cranes in human-dominated paddies. In recent years, the Shengjin Lake NNR Management Center has negotiated with local villages to lease some of the un-harvested paddy fields as foraging sites for the wintering waterbirds. The hooded crane, bean goose *A*. *fabalis*, swan goose and other waterbird species have taken advantage of these artificial foraging sites. Reducing human disturbances in these foraging sites can ensure the success of the initiative. Thirdly, leaving the harvested rice paddies unplowed until cranes migrate north in the coming spring could help cranes to find more food in the unplowed rice paddies [63].
